# Sustainable and efficient protocols for in vitro germination and antioxidants production from seeds of the endangered species *Araucaria araucana*

**DOI:** 10.1186/s43141-021-00280-6

**Published:** 2021-12-09

**Authors:** María C. Gallia, Julieta Echeverri Del Sarto, Guillermina A. Bongiovanni

**Affiliations:** 1 Institute of Research and Development in Process Engineering, Biotechnology and Alternative Energies (PROBIEN, CONICET-UNCo), Buenos Aires 1400, CP 8300 Neuquén, Neuquén Capital Argentina; 2 School of Medicine, National University of Comahue, Luis Toschi Avenue and Arrayanes street, Cipolletti, Río Negro Argentina; 3 School of Agricultural Sciences, National University of Comahue, Route 151 km 12.5, Cinco Saltos, Río Negro Argentina

**Keywords:** Plant cell culture, Biotechnology, Conservation, Antioxidants, Medicinal plants

## Abstract

**Background:**

The Pehuén or Monkey puzzle tree (*Araucaria araucana*) is an evergreen coniferous tree, which has been historically used for social, medicinal, and nutritional purposes. We have recently showed the value of *A. araucana* seeds as a rich source of micronutrients and antioxidants. This endemic species present in a reduced area in Argentina and Chile is endangered because of the low germination rate and the overexploitation of its edible seeds. Thus, the massive extraction of its seeds is ecologically non-viable resulting in limited availability of its active metabolites. However, biotechnological approaches are attractive strategies of production of valuable metabolites and healthy specimens of endangered plants. The aim of this work was to develop a protocol for in vitro production of antioxidants derived from *A. araucana* seeds and to obtain healthy plants by optimized seed germination.

**Results:**

Calli of Pehuén seeds were induced in Murashige and Skoog medium with different combinations of auxins and cytokinins, in light and dark conditions. Callus from embryonic axes developed in medium with 1 mg/l α-naphthaleneacetic acid and 1.5 mg/l 6-benzylaminopurine in light conditions had efficient biomass production, antioxidant activity, high phenolic, and flavonoid content and no cytotoxicity on mammalian cells. Additionally, 100 % germination was obtained in vitro and healthy plants were acclimatized to non-sterile conditions.

**Conclusion:**

In conclusion, in vitro culture of *A. araucana* could provide new and sustainable options for production of its valuable metabolites with possible therapeutic and nutritional uses. Also, optimized plant germination and acclimatization of endangered species can contribute to the preservation of pristine environments.

## Background

A large number of published studies have confirmed that increases of reactive oxygen species (ROS) and reactive nitrogen species (RNS) can bind DNA, lipids, and proteins to attain stability, thereby turning a physiological condition into a pathological state. Thus, reactive oxidizers cause oxidative stress and are associated to development of several diseases in humans, including type 2 diabetes, arthritis, cancer, infertility, ischemia, neurodegeneration, and liver diseases, among others [1, 2]. In this context, experimental and epidemiological data have shown that a diet rich in antioxidants can reduce the risk of the diseases by reducing oxidant-mediated cellular injury and improving human health [1, 2]. The consumption of natural or synthetic antioxidants can mitigate oxidative damage to cells by inhibiting the propagation of oxidative chain reactions [3, 4]. However, synthetic antioxidants have been reported to have negative effects on consumer health, so research on antioxidants from natural resources has now gained considerable attention, mainly plant derived bioactive compounds, such as polyphenols, carotenoids, and vitamins. Polyphenols are synthesized by plants as secondary metabolites and have been extensively studied for their capacity to improve human health. Historically, the beneficial effects of polyphenols have been attributed primarily to their antioxidant capacity and their ability to modulate cellular antioxidant defense mechanisms by inducing the synthesis of detoxification enzymes. Thus, plant extracts with high level of polyphenols can be incorporated in functional foods or as food supplements designed to benefit human health [5, 6].

It is now known that functional foods play an important role in the modern food industry. They represent a promising innovation as they add natural antioxidants to prevent the rancidity caused by oxidation of unsaturated fats, to conserve the nutritive value of food, to improve shelf-life stability of meat products, and to prevent oxidation processes and chronic diseases related to oxidative stress in human [6, 7]. However, the plant species can be driven to extinction due to commercial overexploitation. This overexploitation is accentuated by the climatic change combined with increasing anthropogenic pressures (urbanization sprawl, waste dumping in natural areas, etc.). In this context, the search for efficient methods to produce polyphenols and for sustainable strategies of multiplication of vulnerable plant species is indispensable. Biotechnological production of specialized metabolites in plant cell cultures provides obvious advantages: biomass production is generally quicker than for the whole plant, plant media composition and plant growth conditions can be easily controlled, over-collection of endangered species is avoided, and can be performed in any place. Indeed, in the last decades, huge efforts have been made to develop plant biofactories at an industrial level for the production of bioactive compounds by cultures of undifferentiated cells [8–10]. Furthermore, biotechnological production based on plant cell and organ cultures help not only for the commercial production of several bioactive compounds but also contribute significantly to conservation of medicinal and aromatic plant species, including the anticancer drug paclitaxel (Taxol®; obtained from *Taxus brevifolia*, *T. baccata* and other *Taxus* spp.) (https://phytonbiotech.com/) [11].

Among the various medicinal plant species, *Araucaria araucana* (Molina) K. Koch (Spanish name “Pehuén,” English name “Monkey Puzzle”; family Araucariaceae) has gained considerable relevance in the recent past. This evergreen coniferous tree is endemic of Argentina and Chile and holds strong cultural and spiritual significance for the people who have coexisted with it, historically using it with social, medicinal, and nutritional purposes [12]. Nowadays, humans intensively use *A. araucana* seeds (piñones) because of their nutritional composition and culinary applications in a wide range of regional foods, which are greatly appreciated by tourists. It is estimated that approximately 90 kg/ha and 273 kg/ha of piñones are harvested annually in Argentina and Chile, respectively [13]. However, plants need from 25 to 30 years for optimal development and sexual maturity, although in xeric environment as Argentinian Patagonia sexual differentiation would occur at 75–100 years of age [14]. Piñones are eaten raw, boiled, and toasted and contain antioxidants, polyphenols, flavonoids, and oligoelements like P, K, Mn, Fe, Cu, and Zn [15]. In view of this and many other reasons, *A. araucana* populations have suffered unsustainable exploitation and significant reduction in their distribution area. Pehuén populations continue to be threatened by logging, fires, and extensive livestock use [16, 17]. Additionally, the limited regeneration of Pehuén trees (principally asexual by sprouting from roots) and its low passive dispersion and minor regeneration from seeds produce slow recovery of their populations facing an extremely high risk of extinction in the wild [14, 16]. Furthermore, government programs for conservation purposes that have attempted to obtain seedlings in greenhouses and to improve the dispersal of piñones and the establishment of plantlets in the forest have not yielded efficient results (personal communication). As a consequence of all this, Pehuén is listed as “endangered” species on the Red Data List of the International Union for Conservation of Nature in the category B2ab (ii,iii,v) [16]; it is considered of high conservation value, and it is protected on both sides of the Andes Mountains.

In view of the above reasons and to take advantage of beneficial properties of *A. araucana*, and at the same time promoting conservation of this endangered species, it is important to develop efficient protocols for its multiplication and sustainable methods for obtaining its bioactive metabolites. In this regard, biotechnological tools provide complementary options for production of metabolites from piñones and use of cellular and/or tissue culture techniques for faster multiplication and obtaining highly valued products, such as antioxidants and other bioactive metabolites. In addition, quality plants regenerated in vitro and subsequently acclimated could be used in different forest restoration programs. These biotechnological techniques ensure continuous production of biomass, regardless of seasonal and geographical conditions, without a negative impact on biodiversity [8–10].

In preliminary studies, we noticed the possibility of producing antioxidants from callus of piñones as a sustainable source of its antioxidants [18]. The present work analyses the optimum combination of auxins and cytokinins to obtain high antioxidant content from *A. araucana* seed callus, as an eco-friendly approach to use this valuable resource. Furthermore, the seed germination protocol was optimized to obtain healthy plants of this endangered species.

## Methods

### Chemical reagents

Dulbecco's Modified Eagle Medium (DMEM), antibiotics, reagents, and standards were purchased from Sigma-Aldrich Chemical Co, St. Saint Louis, MO, USA (Argentina office). Solvents HPLC grade were from Thermo Fisher Scientific Inc., Waltham, MA, USA. Fetal bovine serum was purchased from Natocor, Córdoba, Argentina. MilliQ water was used in all experiments. Sterile 0.22-μm PES filters were from GVS Filter Technology, Bologna, Italy. Auxins, cytokinins, Agar, D-Sucrose, and Murashige & Skoog Basal Medium with Vitamins & MES were from PhytoTechnology Laboratories L.L.C, Lenexa, KS, USA. Gibberellic acid GIBERELINA GA_3_ KA® was from S. ANDO Y CÍA SA Buenos Aires, Argentina.

### Vegetable material

**Fig. 1:**
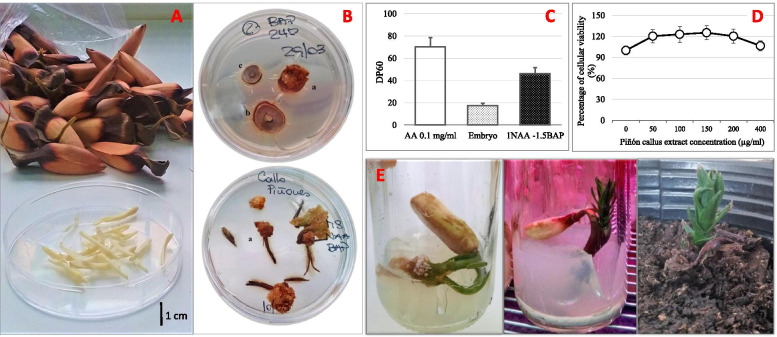
**A** Healthy piñones collected from adults trees located near Villa Pehuenia (Neuquén Province, Argentina) and entire embryos separated used for in vitro culture. **B** Callus formation in piñón slides (up) and isolated embryos (down); where **a** are callus grown in embryonic axis, **b** and **c** are slides of cotyledons and endosperm with no callus formation. Photos correspond to callus 45 days old in 60 mm Petri dishes. **C** Antioxidant capacity of ascorbic acid solution, embryo, and callus obtained in MS medium supplemented with 1 mg/l NAA and 1.5 mg/l BAP, in light conditions. Results obtained by DPPH assay are expressed as percentage inhibition of DPPH radical at 60 s (DP_60_). Different letters indicate statistically significant differences. **D** Effect of different concentrations of callus extract (grown in MS medium with 1.5 mg/l BAP and 1 mg/l NAA in light conditions) on VERO cells viability. Values are expressed as % with respect to control cells (100%). **E** Representative photos of stages of in vitro germination (left), establishment (middle) and acclimatization (right) of *A. araucana* seedlings

Pehuén seeds were harvested from 10 adult trees randomly chosen (mean height 25–35 m and mean DBH 1.0–1.5 m) located in Pehuén forest, near Villa Pehuenia in Neuquén province, Argentina (38° 50′ 02.3″ S 71° 12′ 24.1″W), in April 2017 and 2018. Full and plump piñones were transported to the laboratory. Selected seeds (mean seed length 4–5 cm, mean seed width 1.5–2.0 cm, and mean seed weight 2.3–2.7 g, as shown in Fig. 1A) were kept in plastic bags and stored in a refrigerator at 6 °C, to maintain homogeneous temperature and moisture. In preliminaries experiments [18], callus formation capacity of piñones was tested at different times post-harvesting. Accordingly, all experiments were performed with seeds up to 12 weeks after collection.

### In vitro methodology: callus induction

Seeds were cultured in two forms: slices containing both endosperm and embryo tissues, and entire embryos only. Previously, piñón coat had been removed and both materials (naked seeds and embryos) were separated and disinfected to introduce them in vitro. The sterilization process consisted in submerging the naked seeds or embryos for 2 min in a 70% ethanol solution and then in commercial sodium hypochlorite solution (1.5% active chlorine) for 15 min. Intermediate and final washings were performed with sterile distilled water. Naked seeds were sliced in 5 pieces of 2 mm thickness while embryos were cultured complete.

Callus cultures were established in 60 mm Petri dishes with Murashige and Skoog (MS) medium [19] supplemented with 30 g/l sucrose and 8 g/l agarose, adjusting pH to 5.6 ± 0.2. The medium was fortified with different combinations of auxins and cytokinins in order to select the best conditions for callus growth and antioxidant production. Murashige and Skoog medium with various combinations and concentrations of dichlorophenoxyacetic acid (2,4-D) or α-naphthaleneacetic acid (NAA) used alone or combined with 6-benzylaminopurine (BAP) and gibberellic acid GA_3_ in light (L) and dark conditions (D) was tested. Cultures were kept in a growth chamber at 23 °C under a 16/8 h light/dark photoperiod (white, blue, and red LED lights in a proportion 1:1:2) or in darkness in order to produce callus, which were sub-cultured every 4 weeks. Each Petri dish contained 3-5 embryos or slides and each treatment was replicated five times. The percentage of callus formation at 70 d in each treatment was calculated using the following the formula: % callus formation = number of explants with callus/total number of explants cultured*100.

### Sample preparation

Freeze-dried calli were milled into a fine powder and 1 g of each dried sample were extracted in 12 ml ultrapure water and then kept in the dark for 12 h at 20 °C. In general, dry weight was 7 to 8 times less than fresh weight and no significant differences between treatments were observed. The extracts were centrifuged at 3000 rpm for 10 min and supernatants were filtered through a 10-μm pore nylon filter and stored at − 20 °C until analysis. For comparative purposes, freeze-dried embryo extracts were also obtained.

### Selection of optimum culture conditions

In order to select optimum culture conditions, the following parameters were determined: percentage of callus formation, biomass production, antioxidant power analyzed by FRAP and DPPH assays, total phenolic, and flavonoid contents. Calli obtained under culture conditions resulting in a major percentage of callus formation and biomass production were used to evaluate antioxidant power, phenol, and flavonoid content. For comparative purposes, embryo extracts were also analyzed.

### Ferric reducing antioxidant power (FRAP) assay

Benzie and Strain [20] protocol with minor modifications was employed to study FRAP activity of callus and embryo extracts. An extract aliquot of 50 μl was added to 950 μl of FRAP reagent. FRAP reagent was prepared by mixing 2.5 ml of 10 mM TPTZ (2,4,6-Tri(2-pyridyl)-s-triazine) solution in 40 mM hydrochloric acid with 2.5 ml of 20 mM FeCl_3_.6H_2_O and 25 ml of 300 mM sodium acetate buffer pH 3.6. The reaction mixture was incubated at 37 °C in the dark for 7 min. After that, the absorbance was measured at 595 nm in a T60UV-Visible Spectrophotometer (PG Instruments Limited), and the results were expressed as ascorbic acid equivalents per milligram of callus and embryo (μg AAE/mg DW).

### Free radical scavenging capacity (DPPH assay)

Free radical scavenging ability of the extracts was tested by DPPH (2,2-diphenyl-1-picryl-hydrazyl) radical scavenging assay, based on method as described by Krishnan et al. [21], with some modifications. DPPH radical (0.8 mM) solution in 95% ethanol was prepared. DPPH assay was performed to the best callus induction condition extract. For comparative purposes, embryo extract and ascorbic acid 0.1 mg/ml were also analyzed. An aliquot of 50 μl was mixed with 125 μl of 96% ethanol solution and 625 μl of 1:1 distilled water and ethanol solution. The mix was shaken vigorously and the absorbance was recorded against a blank of ethanol without DPPH.

Kinetics of reactions was monitored at 515 nm on spectrophotometer until no further decrease in absorbance was observed. The percentage of inhibition of DPPH was calculated according to the expression: DP = [(*A*_0_–*A*_*t*_)/*A*_0_]*100 where *A*_0_ is the initial absorbance of DPPH (0% inhibition), and *A*_*t*_ the time “*t*” absorbance after extract mixture has been produced. This time (60 s) was chosen within the interval in which all extracts decay between 35 and 85%.

### Determination of total phenolic content

Total phenolic contents (TPC) of piñón callus and embryo extracts were measured by the Folin–Ciocalteu method according to Soria et al. [22]. Briefly, 50 μl of extract was mixed with 625 μl sodium carbonate (20%, w/v), 200 μl ultrapure water, and 125 μl Folin–Ciocalteu reagent (1 N). After 30 min in the dark, the absorbance was measured at 760 nm in a spectrophotometer. TPC was expressed as μg of gallic acid equivalents per milligrams of dry weight (μg GAE/mg DW).

### Determination of total flavonoids

Total flavonoid contents (TFC) of piñón callus and embryo extracts were determined by the aluminum chloride colorimetric method according to Soria et al. [22]. The extracts (50 μl) were mixed with a solution containing 30 μl of 10% sodium nitrite, 60 μl of 20% aluminum chloride hexahydrate, 200 μl of 1 N NaOH, and 660 μl of water. The absorbance of each sample was recorded at 510 nm on spectrophotometer and compared with those obtained from a standard curve made from quercetin. TFC was expressed as quercetin equivalents per milligram of dry weight (μg QE/mg DW).

### Analysis of biological interaction of extracts: early effects on cell viability

#### Cell culture

African green monkey (*Cercopithecus aethiops*) kidney cells (VERO cell line ATCC n° CCL-81) were cultured in Dulbecco’s Modified Eagle’s medium (DMEM) completed with 10% fetal bovine serum, 100 U/ml penicillin-G, and 40 μg/ml gentamycin sulfate, incubated at 37 °C in a 5% CO_2_ atmosphere [15].

#### Cellular viability

VERO cells were seeded onto 96-well tissue culture test plates at 6000 cells per well. After 24 h post-seeding, the medium was replaced with a fresh one containing callus extract (from the optimum medium chosen) at final concentration of 50, 100, 150, 200, or 400 μg DW/ml of medium or with fresh medium only (control). Viable cells were stained with 0.5% crystal violet in 50% methanol in water solution, for 15 min. After being washed with 50% methanol three times, the stained cells were solubilized with a solution containing 0.1 M sodium citrate, pH 5.4, and 20% methanol. The absorbance of each well was read at 630 nm with a Rayto RT-2100C microElisa reader (Rayto Life and Analytical Sciences Co., Ltd. China). The viability percentage was defined as the relative absorbance of treated versus untreated control cells (100%), and represents the mean ± SD of two independent experiments.

### In vitro germination and plantlets acclimatization

#### Germination

In order to evaluate germination, two types of piñones were used: piñones with whole coat and peeled piñones. After washing them, they were left to soak 12 h in water. Then, disinfection was performed with 70% (v/v) ethanol for 2 min, followed by 1.5% (w/v) sodium hypochlorite for 15 min and washed three times with sterile distilled water. Seeds were incubated in agarized (0.8% w/v) MS medium with 3% (w/v) sucrose, at pH 5.6. Cultures were maintained at 23 ± 2 °C, under 16/8 h light/dark photoperiod using LED lights (white, blue and red in a proportion 1:1:2). The experiment was carried out with fifteen randomly selected seeds per treatment and was repeated five times.

#### Acclimatization

Plantlets (2 month-old) were carefully removed from the glass culture vessels; roots were washed with sterile water to remove medium and then transferred into sterile pots filled with sterilized soil and sand (2:1 v/v). Soil from Pehuén forest, previously collected in Villa Pehuenia (38° 51′40.140′ S 71° 3′ 51.264′ W) was used. Plantlets were acclimated for 7 weeks under culture room conditions. In the first 2 weeks, each pot was covered with a polyethylene bag, which was perforated in the third week to allow air exchange. Plantlets were grown for 1 week with the polyethylene bag perforated. Then, polyethylene bags were removed and plantlets were watered every 2 days for 2 weeks with distilled water and with tap water for the next 2 weeks. Later, plants were moved to a greenhouse in 16-cm-wide polypropylene pots filled with soil from Pehuén forest. Thirty days after transplanting the plantlets to the greenhouse, the percentage of seedling survival was evaluated.

### Statistical analysis

All experiments were conducted at least two times. For statistical analysis, all assays were performed in triplicate, and quantitative data were expressed as mean of two experiments ± standard deviation. Data were analyzed with InfoStat version 2020. Student’s *t* tests were carried out to determinate differences between treatments, where *P* value < 0.05 was considered statistically different.

## Results

### Selection of optimum culture conditions

#### In vitro callus induction from piñón

For piñón callus induction, different combinations of the auxins NAA and 2,4-D with BAP and GA_3_ were tested. Callus cultures from piñones developed after 10–14 days, only in the embryonic axis, not in cotyledons or endosperm. Figure 1B (up) shows that the slide containing embryonic axes (a) developed callus in this tissue, contrary to what happened with those slides containing parts of cotyledons and endosperm (b and c). This observation can be clearly seen also in Fig. 1B (down), when isolated embryos were cultured. Then, embryos were used to callus induction. The results showed callus formation when the auxin NAA was used under light or dark conditions while there was low or null callus formation using 2,4-D (Table 1). The best conditions of piñón callus induction was in MS medium supplemented with 1 mg/l NAA and 1.5 mg/l BAP, grown in light conditions with 57.1% of callus formation (Table 1, treatment 7). A decreased biomass production was detected when GA_3_ was added to the best induction condition. Calli developed in 2 mg/l 2,4-D and 1 mg/l BAP, and 1 mg/l NAA and 2 mg/l BAP in light conditions (Table 1, treatment 6 and 9, respectively) showed low percentage of formation and weight (12.6 and 3.5 mg per callus, respectively), so they were not taken into account for the next measurements.

**Table 1:** Effect of different combinations and concentrations of growth regulators in light (L) and dark (D) conditions on callus formation from piñón embryo

Treatment	2,4-D	NAA	BAP	GA_**3**_	% callus	DW^a^	FRAP	TPC	TFC	FRAP 100^§^	TPC 100^§^	TFC 100^§^
	mg/l	mg/l	mg/l	mg/l	% callus	(mg)	μg AAE/mg DW	μg GAE/mg DW	μg QE/mg DW	μg AAE	μg GAE	μg QE
**Light**												
**1**	1	0	0	0	ND							
**2**	2	0	0	0	ND							
**3**	0	0	1	0	ND							
**4**	0	1	1	0	ND							
**5**	1	0	1	0	ND							
**6**	2	0	1	0	6	12.60^b^						
**7**	0	1	1.5	0	57	40.60	10.50*	9.67*	12.00*	20828.63*	19175.57*	23804.15*
**8**	0	1	1.5	0.5	44	19.60	10.17*	9.33*	12.17*	7515.50*	6899.48	8993.96*
**9**	0	1	2	0	15	3.50^b^						
**10**	0	2	2	0	ND							
**11**	1	0	2	0	ND							
**12**	2	0	2	0	ND							
**Dark**												
**13**	1	0	0	0	ND							
**14**	2	0	0	0	ND							
**15**	0	0	1	0	33	19.90	9.50*	6.50*	6.50*	5078.90*	3475.04*	3475.04*
**16**	0	1	1	0	ND							
**17**	1	0	1	0	ND							
**18**	2	0	1	0	ND							
**19**	0	1	1.5	0	38	14.70	8.50*	9.00*	23.17*	4069.96*	4309.37	11092.64*
**20**	0	1	1.5	0.5	33	6.02	3.33 ± 1.34	5.83*	15.50	567.62*	993.34*	2639.45*
**21**	0	1	2	0	25	35.70	5.50*	14.33*	8.50*	4207.67*	10965.44*	6502.76*
**22**	0	2	2	0	ND							
**23**	1	0	2	0	ND							
**24**	2	0	2	0	ND							
**None**	Embryo					54	1,8 ± 0,2	0,98 ± 0,27	16,07 ± 1,03	9720	5292	86778

Values represent the mean of three independent experiment performed in duplicate ± SD. a: mean dry weight of each embryo or callus. b: not analyzed samples since low callus induction. *Significant differences P ≤ 0.05. For a better visualization of the results, the SDs were not included for all the values in the table. However, all SDs were less than 15%, except for FRAP of treatment 20. Bold values: increased efficiency with respect to embryo. §: calculated value to 100 embryos

#### In vitro production of antioxidants

In this work, we analyzed antioxidant activity of piñón callus extracts by FRAP assays. Additionally, DPPH assay was performed to the best callus induction condition (Table 1, treatment 7). The results showed that callus developed in both light and dark conditions had higher antioxidant capacity compared to embryo, except those obtained in MS medium supplemented with 1 mg/l NAA, 1.5 mg/l BAP, and 0.5 mg/l GA_3_, in dark conditions (Table 1, treatment 20). Callus obtained in MS medium supplemented with 1 mg/l NAA and 1.5 mg/l BAP in light conditions had 10.5 μg AAE/mg DW and 43% of inhibition of radical DPPH (DP_60_ in Fig. 1C), while embryo had significantly lower antioxidant power (1.2 μg AAE/mg DW and 17 % inhibition of radical DPPH (Fig. 1C).

#### Determination of total phenolic and flavonoid contents

It has been suggested that in optimization of metabolite production, the first stage consists in choosing the appropriate culture conditions to obtain higher percentage of induction and maintenance [23]. For this reason, embryos under treatments that induced major callus formation were selected to continue the studies and compare antioxidant activity.

The callus extracts that were selected (treatment 6 and 9 were discarded) had significantly more TPC than embryo extracts, between 5 and 14 times. Extracts of piñón embryo had 0.98 μg GAE/mg DW while callus under the best induction condition had 9.67 μg GAE/mg DW.

Under our cell culture conditions, callus grown in similar medium had lower TPC in the dark than those grown in light conditions. On the other hand, piñón callus grown in dark conditions had significantly higher TFC than those obtained in light. In this regard, when TFC of callus is compared with that of the whole embryo, only treatment 19 showed significantly higher values (23.17 vs. 16.07 μg QE/mg DW, Table 1). Additionally, the antioxidant efficiency was determined by comparing antioxidant production of 100 embryos with antioxidant production of calli from 100 embryos. So, only treatment 7 (1 mg/l NAA and 1.5 mg/l BAP, in light conditions) was highly efficient.

#### Biological test for callus extracts

Early effects on VERO cells viability were evaluated in order to discard toxic effects. It was found that extract from callus grown in the optimum medium chosen (MS medium with 1 mg/l NAA and 1.5 mg/l BAP in light conditions) did not decrease cellular viability at any of the concentrations used after 2 hours of incubation (Fig. 1D). As a result, in vitro culture of piñón embryo cells in these conditions would not add any toxic substance that could decrease VERO cells viability.

#### In vitro germination and acclimatization

In this work, piñón germination was improved with a previous imbibition treatment, by pulling off the piñón coat and with incubation at 23 °C. Thus, a 100% in vitro germination was achieved at 6 d and healthy plants were obtained (Fig. 1E). Piñón scarification allowed water to enter and it unchained metabolic and physiological mechanism of germination. Accordingly, piñón-soaking pretreatment combined with efficient and maintained disinfection could be the main factor to obtain 100% of germination because imbibition triggers metabolic and physiological mechanisms of germination.

Plantlets removed from the culture medium and washed with sterile water were well acclimated to new substrate. Furthermore, all the plantlets acclimatized in the greenhouse in non-sterile conditions with soil collected from Pehuén forest showed development of new leaves after 2 weeks (Fig. 1E).

## Discussion

*A. araucana* is an evergreen coniferous tree, which has been historically used for social, medicinal and nutritional purposes [12]. It is an endemic species of high conservation value, present within a reduced area in Argentina and Chile and protected by both countries. Nowadays, the intensive use of its seeds due its nutritional composition, the reduction of its distribution area for agricultural and forestry use, and the limited regeneration from seeds produce slow recovery of their populations facing an extremely high risk of extinction in the wild [14, 16]. Thus, germination and production of its valuable metabolites through in vitro culture are recognized as possible alternatives for the multiplication, conservation, and utilization of endangered species [9, 24, 25].

To obtain secondary metabolites, we developed nonembryogenic calli with homogenous and dedifferentiated cells, according to Cardemil and Jordan [26], who observed that only embryonic cells of *A. araucana* had the capacity to form callus and to induce callus in the megagametophyte.

In this regard, it is well known that plant hormones can act as elicitors through interaction with specific target tissues, inducing genetic and physiological responses in plants. In particular, auxins and cytokinins signaling pathways have been related to promotion of callus induction [9]. This study revealed that the MS medium enriched with 1 mg/l NAA and 1.5 mg/l BAP was observed to be optimal for the induction and growth of callus, in light conditions. Compared to the antioxidant activity found in the embryo, this biomass produced 2.14 times higher antioxidant activity than one embryo. This result suggests that the biomass of piñones produced in vitro would be an interesting nutraceutical candidate for human consumption. Additionally, our previous results [15] showed that pulp (edible part composed by embryo and endosperm; mean weight 1.8 g per piñón) of one piñón contains 1.55 mg AAE, while 0.208 mg AAE can be provided from callus of one embryo. However, 540 days (18 months) are necessary to obtain one matured seed, while in vitro production is continuous (24/7). Therefore, although plant in vitro (tissue culture) techniques are time-consuming because several parameters have to be tested to obtain an optimum system for metabolite production, the overall production time was significantly shorter than the growth cycle of a whole plant. Certainly, plant cell cultures have been described to be more efficient in antioxidants extraction than plant collection from nature, so they represent alternative sources for the easy and scalable production of secondary metabolites [10, 27]. In that way, this in vitro method, independent of climatic and geographical conditions, could provide continuous, sustainable, and viable production of natural antioxidants derived from *A. araucana*. Furthermore, in vitro plant cultures offer the possibility to increase the synthesis of selected secondary metabolites by using elicitors, by manipulation of metabolic pathways and metabolic engineering, as well as by biotransformation. In addition, production can be scaled up in bioreactors [10, 27]. As it occurs in several vegetable foods, polyphenol content is highly related to antioxidant activity, and it is well known that light is a physical factor, which stimulates the secondary metabolites biosynthesis, including phenolic compounds, in in vivo and in vitro conditions. Additionally, phenolic content and antioxidant activity in cell cultures varies largely, depending upon light wavelength, plant species, and culture type [28, 29]. Our results suggest that polyphenol content increased during in vitro culture conditions, mainly under light conditions. An increase in the production of polyphenols was also described in callus obtained from seeds of other species [30]. These results can be explained by the occurrence of more phenolic content in the embryonic axes (from which we obtained piñón callus) than in cotyledons, as it occurs in several seeds [31, 32]. Indeed, the presence of polyphenols in embryonic axes has been closely related to seed viability because of their role in scavenging ROS and preventing aging processes [33]. Contrarily, flavonoid content of callus was lower or similar (mainly in dark conditions) when compared with that of the whole embryo. Therefore, results suggest that the greater antioxidant power of callus compared to that of embryos may be related to a higher synthesis of non-flavonoids polyphenolic compounds under in vitro conditions, mainly in light. Thus, the combination of 1 mg/l auxin NAA and 1.5 mg/l cytokinin BAP promoted the initiation and maintenance of in vitro culture of piñón embryo cells and stimulated biosynthetic pathways for the production of high-value metabolites for nutrition and health. Thus, this biotechnological method, based on in vitro culture, is currently being applied at the industrial level for the production of several phytochemicals with similar or superior yields to those of plants crops. Furthermore, plant cell culture is the only economically viable way of producing some high-value metabolites from endangered plants. Some examples include arbutin, berberine, and shikonin manufactured by Mitsui Chemicals, Inc., ginseng by Nitto Denko Corporation, and paclitaxel by Phyton Biotech Inc. [11].

Considering the nutraceutical potential of piñón callus, cytotoxicity of the extract was determined. As expected, no early cytotoxic effects were found in VERO cells exposed to piñón callus extract, such as it was reported by Gallia et al. [15] with piñón embryo, endosperm, and coat. This results agree with the historical medicinal and nutritional uses of these seeds by regional communities [12] and other research about cytoprotective effects of phenolic extracts of species of the genus *Araucaria* [34, 35].

Indeed, socio-cultural factors (ancient and actual) have also contributed, together with environmental factors, both to the preservation of these living fossils and to their current state of degradation and fragmentation. Sexual reproduction of *A. araucana* occurs mainly in areas with better state of conservation, as observed in protected areas such as the Lanin National Park (https://www.argentina.gob.ar/parquesnacionales/lanin). In contrast, in those populations that inhabit areas with disturbances, such as livestock and human pressures, asexual reproduction by root suckering predominates [35]. This behavior could decrease genetic variability, distribution area, and adaptability to new environmental conditions. Some authors have suggested that modern technologies like in vitro production of planting material and secondary metabolites could be used for faster multiplication and large scale production [24, 25]. Therefore, various approaches can be taken to protect *A. araucana* through in vitro culture. For example, the planting material could be used for reforestation of areas that have been exploited for agricultural-livestock use in which the density of trees has decreased. This idea is in keeping with the fact that following seed germination and raising nursery, transplantation of such seedlings within and outside the forest range carried out by local communities has been one of the reasons of community involvement in conservation of the target species [12]. Furthermore, native plantations for economic purposes in nearby areas could mitigate the effect of habitat loss and fragmentation. This will ensure conservation of other native species and at the same time allow sustainable harvesting of the relevant species, as has been demonstrated in *A. angustifolia* plantations [36]. 

In *A. araucana* seeds, the embryo keeps its metabolic activity throughout ontogeny and if the environmental conditions are optimum, they could germinate rapidly after dispersion event [14]. However, piñones are recalcitrant seeds and have an impermeable seminal tegument that prevents water entering, and it generates mechanic resistance to embryo growth, so the germination event has some limitations. In this study, after 6 days, 100% in vitro germination was achieved with a previous imbibition treatment, scarification, and incubation at 23 °C. In natural conditions, scarification could be caused by the knock of piñones onto the rocky soil, the grazing by animals, fungi and bacteria, or water and snow dispersion [14, 26], and germination could occur during the wet season. A previous study has reported that imbibition treatment effectively enhance germination rate and percentage in seeds of *A. araucana* [14]. However, in that study, the germination percentage did not exceed 50% germination (just obtained after 15 days) because no sterile culture conditions were used and seeds quickly rotted. Our results show that the in vitro germination protocol developed in this study, despite being more expensive and less simple, is efficient. Furthermore, such in vitro germinated plants could be further used for micropropagation and/or directly planted into its natural habitat; such strategies have been successfully reported for numerous endangered species with medicinal use [25, 37, 38].

## Conclusions

The present research provides a conservationist protocol for use and multiplication of endangered species *Araucaria araucana*. Secondary metabolites of great interest for health and vigorous plants can be obtained preserving this species and its environment. Callus developed in the embryonic axes of piñón produced high values of antioxidant activity, phenolic and flavonoid content, with non-toxic effect in mammalian cells. This work is the first step towards obtaining valuable secondary metabolites from *A. araucana*, with possible therapeutic and nutritional uses following a sustainable, efficient, and reliable procedure. In addition, in vitro germination of piñones is considered a promising strategy for the regeneration of the species.

## Data Availability

Not applicable.

## Abbreviations

DMEM: Dulbecco’s Modified Eagle Medium
BAP: 6-Benzylaminopurine
NAA: Naphthalene acetic acid
2,4-D: Dichlorophenoxyacetic acid
GA_3_: Gibberellic acid
GAE: Gallic acid equivalents
AAE: Ascorbic acid equivalents
QE: Quercetin equivalents
DW: Dry weight
TPC: Total phenolic content
TFC: Total flavonoid content
